# *‘No Austrian Mother Does This to Sleep Without a Baby!’* Postnatal Acculturative Stress and ‘Doing the Month’ Among East Asian Women in Austria: Revisiting Acculturation Theories From a Qualitative Perspective

**DOI:** 10.3389/fpsyg.2020.00977

**Published:** 2020-05-14

**Authors:** Yuki Seidler, Radhika Seiler-Ramadas, Michael Kundi

**Affiliations:** ^1^Center for Public Health, Medical University of Vienna, Vienna, Austria; ^2^Department of Development Studies, University of Vienna, Vienna, Austria; ^3^Center for Health and Migration, Vienna, Austria

**Keywords:** acculturative stress, lived experience, postnatal care, immigrant, East Asian women, Austria, interpretative phenomenological analysis, qualitative methods

## Abstract

Acculturative stress is a phenomenon describing negative emotions experienced by immigrants in their socio-cultural and psychological adaptation process to the host society’s dominant culture and its population. Acculturative stress is assumed to be one the reasons for higher prevalence of postnatal depression among immigrant women compared to non-immigrant women. Theories and models of acculturation and coping strategies suggest that certain cultural orientations or behaviors could mitigate acculturative stress and postnatal depression. Nevertheless, quantitative studies applying these theories have so far revealed inconsistent results. Given this background, we ask: what can a qualitative study of immigrant women’s postnatal experiences tell us about the interrelationships between immigrant mothers’ acculturation behaviors or cultural orientations, and maternal psychological health? Particularly, we explore the postnatal experiences of Chinese and Japanese women who gave birth in Austria, focusing on their experiences and behaviors influenced by their heritage culture’s postnatal practices (*zuò yuè zi* and *satogaeri*). Theoretically, we apply Berry’s acculturation model through a focus on what we call ‘Postnatal Acculturative Stress’ (PAS). By doing so, we identify factors that prevent or mitigate PAS. Another aim of this article is to critically reassess Berry’s model in the context of postnatal care and maternal psychological health. Data were analyzed using a combination of deductive and inductive method through the application of directed content analysis and phenomenological approach. Women’s postnatal experiences were summarized as an ‘unexpected solitary struggle in the midst of dual identity change’ in four specific domains: postnatal rest and diet, social support, feelings toward significant others and identity. Preventive and mitigating factors against PAS included trust (in self and one’s health beliefs) and mutual respectful relationships with and between the significant others. The application of Berry’s acculturation model provided a useful framework of analysis. Nevertheless, the multifarious complexity involved in the process of acculturation as well as different power dynamics in the family and healthcare settings makes it difficult to draw causal relationships between certain acculturation behaviors or cultural orientations with specific health outcomes. Health professionals should be aware of the complex psychosocial processes, contexts as well as social environment that shape immigrants’ acculturative behaviors.

## Introduction

In cultural psychology, *acculturative stress* is a phenomenon understood as negative emotions experienced by immigrants in their socio-cultural and psychologically adaptive process to the host society’s dominant culture and its population (Berry, 1997). Originating in Lazarus and Folkman’s (1984) psychological theory of ‘stress, appraisal and coping,’ the concept differs from the popular term of *culture shock* as it involves not only the short-term momentum of *shock* resulting from an encounter with a new culture and its people but a longer-term process of appraisal and coping with negative experiences (Berry, 1997). Several quantitative studies report acculturative stress to be one of the major risk factors for postnatal depression and related symptoms among non-Western immigrant women in Western countries (Zeider et al., 2015; Alhasanat-Khalil et al., 2018; Dennis et al., 2018). Cultural issues are frequently identified as one of the reasons for a sustained higher prevalence of postnatal depression and symptoms among immigrant women compared to non-immigrant women (Collins et al., 2011; Eastwood et al., 2011; Almeida et al., 2013; Falah-Hassani et al., 2015).

Literature suggests that certain types of cultural orientations (Alhasanat and Giurgescu, 2017; Sharapova and Goguikian Ratcliff, 2018) and specific postnatal cultural practices (Fung and Dennis, 2010) could be associated with maternal depression and mood disorders. A number of studies — many of them conducted among immigrant women of Hispanic origins in the United States (U.S.) — have applied Berry’s (1997; 2005) acculturation model to identify if certain acculturative behavior is associated with postnatal depressions. In Berry’s (1997; 2005) model, immigrants typically resort to the following four types of behavior when encountering acculturative stress: (1) assimilation (deserting ones cultural heritage and shifting toward the norms of host culture); (2) integration/biculturalism (maintaining ones cultural heritage and at the same time adopting the norms of the host culture); (3) separation (maintaining ones cultural heritage without adopting the norms of the host culture); and (4) marginalization (maintaining or adopting neither of the cultures) (Berry, 1997, 2005; Kuo, 2014). There are naturally other models that explore acculturation and coping strategies. Kuo (2014), for example, compares Berry’s model with three other such models: resilience-based coping model (Castro and Murray, 2010), a grounded theory-based model (Yakushko, 2010) and ‘stress–mediation–outcome theory’ (Cervantes and Castro, 1985). While all three could be used in exploring the interrelationships between acculturative behaviors, coping strategies and immigrants’ health, we consider Berry’s model to be the most suitable in investigating into the maternal psychological health of immigrant women. Unlike other models, Berry’s model specifically focuses on the coping strategies against acculturative stress and not just against any stress (Kuo, 2014). This is particularly important as birth is physiologically universal but organized in ‘culturally’ different ways in different societies (Jordan, 1978/1993). There are ample occasions for immigrant women to encounter various kinds of acculturative stress when giving birth in a foreign land. Berry’s model is also unique in that it incorporates the acculturation behavior and attitudes of not only the immigrants but also those of the host population (Kuo, 2014). For example, his model predicts that the openness of the host population to immigrants’ culture is a critical factor in realizing the integration strategy, which in turn has a positive impact on immigrants’ psychological health (Berry, 1997, Berry, 2006). As birth has become highly institutionalized and medicalized in Western countries, it is almost impossible for an immigrant woman to give birth without having intensive interactions with the host country’s healthcare system and health personnel. Thus, the incorporation of the host population’s attitude and behavior is essential in exploring the maternal health of immigrant women.

Quantitative studies applying Berry’s model of acculturation strategies, however, do not always make the types and nature of acculturative stress faced by the women explicit, nor incorporate the attitudes and behavior of the host population. Related studies applying Berry’s model to predict postnatal depression have so far revealed inconsistent results making it difficult to identify mitigating factors against maternal depression related to acculturative stress. For example, greater alignment to heritage culture (*separation*) was found to be protective against postnatal depression among the Pacific Islanders in New Zealand (Abbott and Williams, 2006) while the opposite was found among the immigrant women in Switzerland (Sharapova and Goguikian Ratcliff, 2018). In the New Zealand study, *marginalization* was associated with higher, and *integration* with lower depression risks but separation was also identified as a protective factor (Abbott and Williams, 2006). Among the women of Hispanic origin in the U.S., higher acculturation to the U.S. culture was associated with higher risks for postnatal depression and adherence to heritage cultural practices as protective (Alhasanat and Giurgescu, 2017).

Literature suggests adherence to heritage culture’s postnatal practices have both positive and negative effects on the postnatal mental health of Asian immigrant women as well (Fung and Dennis, 2010). In many Asian cultures, the postnatal period — up to around 40 days after birth — is considered as a time for the mothers to nurture themselves and to enjoy a legitimate time of physical rest while mentally preparing for the transition to motherhood (Son, 2016). The most relevant to our current study is the custom of *zuò yuè zi* widely practiced among the Chinese-speaking population and *satogaeri bunben* practiced by the Japanese. *Zuò yuè zi* in Chinese literally means ‘sitting in for the first month’ or ‘doing the month’ (Cheung, 1997; Holroyd et al., 1997). Mothers are confined to the house for up to 40 days after birth with a special diet and modified behavior in order to rest and fully recover from birth. It is practiced widely by both urban and rural populations of various socio-economic classes among the Chinese-speaking population within and outside their countries of origin. Typical practices maintained by Chinese mothers interviewed in Scotland (Cheung, 1997) and Australia (Matthey et al., 2002) included: a special postnatal diet; house confinement; avoiding anything cold; freedom from domestic duties and abstinence from recreational activities. *Zuò yuè zi* is usually done with the support from female relatives: especially mothers-in-law (Wong and Fisher, 2009). *Satogaeri* in Japanese means ‘returning to old home’ and *bunben* ‘to give birth’. Typically, Japanese mothers go back to their own parents’ house 1–2 months prior to birth and stay 1–2 months after birth to rest properly and receive physical (housekeeping chores) and mental (learning about childbearing or not being alone) support from their own mothers (Yoshida et al., 2001; Ito and Sharts-Hopko, 2002). Although this means that women live separately from their husbands for several months, it is still a widely practiced custom in contemporary Japan (Yoshida et al., 2001; Kitamura et al., 2006; Iseki and Ohashi, 2014; Takahashi and Tamakoshi, 2014). A modern ‘reversed’ form of *satogaeri* in which mothers stay at her daughters’ home is practiced not only in Japan but among some of the Japanese women living overseas (Yoshida et al., 2001). Unlike the *zuò yuè zi*, the practice of *satogaeri bunben* (hereafter referred to as *satogaeri*) has less rules and the primal objective is to rest, eat well and be freed from household duties (Yoshida et al., 2001). In both cases, however, the element of strengthened social support from female relatives and peers is an important function of the practice. Similar to the aforementioned studies on cultural orientations and postnatal depression, adherence to *zuò yuè zi* or *satogaeri* among Chinese and Japanese women living in Western countries reveal both protective and negative effects on depression (Matthey et al., 2002; Bina, 2008). Literature suggests that such practices could be both a cause of and buffer against acculturative stress (Cheung, 2002a, b; Taniguchi and Baruffi, 2007; DeSouza, 2014). Some argue that the positive effects are mainly due to the increased social support acquired through the practices (Matthey et al., 2002; Fung and Dennis, 2010). Nevertheless, the reasons for the above mixed results as well as the complex associations between cultural orientations, postnatal rituals, social support and maternal psychological health are not well understood. In fact, despite the relatively large amount of literature on the topic, very little has been done to investigate into the ‘lived experiences’ (Smith et al., 2009, p. 33) of acculturative stress during the postnatal period from immigrant women’s perspectives, and what could contribute in preventing the stress. Moreover, studies that measure and apply acculturative stress quantitatively without incorporating these subjective perspectives on acculturation and acculturative stress seem to encounter fundamental methodological and epistemological problems, which have also been identified by critics on acculturation studies (Chirkov, 2009a, b; Rudmin, 2009).

Given this background, we ask the following question: what can a qualitative study of immigrant women’s postnatal experiences tell us about the interrelationships between immigrant mothers’ acculturation behaviors or cultural orientations, and maternal psychological health? Particularly, we will explore the postnatal experiences of Chinese and Japanese women who gave birth in Austria, focusing on their experiences and behaviors influenced by their heritage culture’s postnatal practices (*zuò yuè zi* and *satogaeri*). Theoretically, we apply Berry’s acculturation model through a focus on what we call ‘Postnatal Acculturative Stress’ (PAS). By doing so, we identify factors that prevent or mitigate PAS. Another aim of this article is to critically reassess Berry’s model in the context of postnatal care and maternal psychological health.

In this paper, acculturative stress is defined as negative emotions experienced by an individual who is aware that such emotions are caused by intercultural interactions and cultural differences (Berry, 2005). We understand enculturative stress — defined here as a stress caused by interpersonal conflict between people from the same cultural heritage (Zeider et al., 2015) —also as a type of acculturative stress. Postnatal Acculturative Stress (hereafter PAS) in our study is defined as acculturative stresses experienced immediately after and approximately up to 6 months after birth that are directly or indirectly related to the birth experience in Austria. Cultures, in our study, are defined in a cognitive sense as common systems of knowledge or belief that are learned and internalized through shared practices and repeated interactions among a group of people (Pool and Geissler, 2005, p. 8). We understand cultures as dynamic ideational systems that one can refer to as guidance for behavior which is considered appropriate and acceptable in a given cultural context and time (Goodenough quoted in Keesing, 1974). Ethnicity in this paper is defined as a fluid concept referring to ‘the group to which people belong, and/or are perceived to belong,’ due to shared attributes such as ‘geographical and ancestral origins, cultural traditions and languages’ (Bhopal, 2004, p. 441).

## Methodology

### Design

Our qualitative research is inspired by existing theories and models. We therefore used a deductive approach called ‘directed content analysis’— a qualitative method primarily used to extend or validate existing theoretical concepts (Hsieh and Shannon, 2005). This was combined with an inductive approach inspired by Heideggerian philosophy of phenomenology (van Manen, 2017). We consider this phenomenological approach appropriate as the phenomenon of acculturative stress is well known and used, but very little is known on what it actually means to postnatal immigrants’ psychological health in the specific context of Austria (van Manen, 2017). We believe that the quantitative methodologies and methods applied in mainstream acculturation psychology are not sufficient in fully capturing the phenomenon of acculturation which the immigrant themselves may not be aware of (van Manen, 2017). Acculturation is a fluid and an interactive process that is highly contextual in nature, making it difficult to develop appropriate survey questionnaires (Chirkov, 2009a, b). Quantitative studies on acculturation are often based on the positivist philosophy and assumption that there is a ‘universal covering law’ to the phenomenon of acculturation and acculturative stress that is ‘predictable’ and ‘controllable’ (Chirkov, 2009a, b). In these studies, acculturative stress is applied as a ‘measurable variable’ without clearly defining what it actually is, and how it could differ in context and time (Rudmin, 2009). To overcome these epistemological constraints, we chose the combined qualitative methods. Our aim was not only to understand and describe the phenomenon in concern but to critically appraise and reflect on our understanding of existing theories that could better inform practices and future research (Salsberry, 1989).

### Rationale for the Sampling Population and Study Location

Chinese and Japanese women in Austria provide a unique case. First, East Asians have distinct cultures from that of the host population. The Chinese and the Japanese especially share many cultural aspects (such as family relations) and health practices (such as ‘doing the month’) rooted in Confucianism and common medical history (Lock, 1980, 1985). They are also one of the minority ethnic immigrant populations in Austria. Women with ethnic minority status are known to be at particular risk of maternal depression (Robinson et al., 2016). This justifies our decision for selecting these two populations. Second, most studies so far have been conducted in the context of English-speaking multicultural societies such as Australia, Canada, or the United States (U.S). The degree of openness and the spirit of inclusiveness transmitted by the people of the host society have a strong influence on immigrants’ experiences of acculturative stress (Kartal and Kiropoulos, 2016). As multiculturalism is neither a shared norm nor a government policy in Austria, the German-speaking context here provides a unique perspective. Finally, including the two subgroups of the East Asian population — the Chinese and Japanese — enables us to explore homogeneities and heterogeneities between and among these groups that share common medical and health traditions (Lock, 1980, 1985).

### Context in Vienna, Austria

This study was conducted in the capital city of Vienna, which has the highest number and proportion of immigrants in the country (Statistics Austria, 2018). In Austria, 99% of births take place in hospitals. In most cases, physicians carry out obstetric care, assisted by midwives and nurses (Bodner-Adler et al., 2017; Luegmair et al., 2018). A wide range of services offered by public midwives, lactation consultants and social workers are available in Vienna, such as prenatal classes, consultations, counseling, and mother-baby groups. Women can request a limited number of home visits from public midwives, or hire private midwives for more comprehensive home-based or individualized care. Multilingual services are available but are limited to the major immigrant languages of Arabic, Bosnian, Croatian, Serbian, Turkish, English, and French (City of Vienna, 2015).

East Asians (defined as those born in China, Hong Kong, Japan, Macau, Mongolia, and South and North Korea) in Austria make up only 1% (or approximately 25,000) of the entire non-Austrian-born population. The actual number is unknown and estimated to be much higher, but they are clearly one of the minority ethnic groups. The biggest East Asian sub-groups are the Chinese (68%) followed by the Japanese (15%), with more than half of them living in Vienna. In 2017, around 5,800 Chinese women and 1,300 Japanese women were officially registered in Vienna. More than 50% were in their reproductive ages between 18 and 49 years old (Statistics Austria, 2018).

### Sampling and Recruitment

We recruited Chinese and Japanese women living in Vienna through purposive sampling (Patton, 1990; Smith and Osborn, 2008). The first author, YS, contacted the leaders of Mandarin-, Cantonese-, and Japanese native-speaking groups in Vienna through e-mail and telephone calls. The purpose of these self-organized community groups is to pass on the parents’ (or one of the parent’s) cultural and linguistic heritage to their children by organizing regular meetings with other families who speak the same mother tongue. This sampling frame ensured that the women had some interest in maintaining their heritage culture, and were thus able to talk about experiences related to acculturation or enculturation, or both. The interviewer asked the leaders of the three groups for individual interviews and also asked them to introduce her to other Chinese-speaking or Japanese mothers who fit into the inclusion criteria: (1) identified herself as having a Japanese or Chinese heritage (and not based on their nationalities or place of birth); (2) had given birth in an urban setting in or around Vienna (in a city or town with more than 10,000 populations) no longer than 5 years prior to the interview; (3) was directly or indirectly related to the language groups; (4) spoke good English or Japanese sufficiently enough to manage the interview without an interpreter and (5) could consent to the study herself. Since not all Chinese-speaking participants were born in mainland China, they are referred to as ‘Chinese-speaking’ women in this study. All Japanese participants were born in Japan and are thus referred to as ‘Japanese’ women. Two Chinese-speaking women declined to participate due to time constraints and having small children. Data saturation was achieved when no new codes were identified from the last two interviews (including follow-up interviews) that led to: (1) a development of a new subordinate theme related to the research question; and (2) the substantial expansion of the four categories used in Berry’s model of acculturation strategies. The final sample included four Chinese-speaking women and six Japanese women. This sample size was considered sufficient to generate meaningful knowledge grounded in the data that answered our research questions (Patton, 1990).

### Ethical Consideration

Consent forms together with a detailed information sheet clarifying the aim of the study, anonymity and confidentiality were prepared in both English and Japanese. All women in this study read the information sheet, understood how anonymity and confidentiality would be maintained and signed the consent form. The participants were informed that the aim of the study was to explore the perinatal experiences of East Asian women in Austria, that the researchers received no funding and that the study was part of YS’s degree program. No participants received remuneration of any kind for participating. Real names were replaced with pseudonyms before sharing the data with the second and the third authors. Ethical approval was obtained from the London School of Hygiene and Tropical Medicine, Research Ethics Committee (010/31).

### Data Collection

Semi-structured ‘biographic-narrative interviews’ (Wengraf, 2001) were conducted by YS in Japanese or English with an occasional mix of German and Chinese words. All Japanese women spoke Japanese. One Chinese-speaking woman was a native English speaker and the other three women spoke fluent English and German. YS is a bilingual Japanese and English speaker with an advanced knowledge of German and little knowledge of Chinese. All interviews started with a single (narrative) question of ‘How did you end up giving birth in Austria?’ with the intention to capture as many life-stories as possible that were related to our topic of interest and for free themes to emerge from the women’s perspective (Wengraf, 2001). This was followed by subsequent thematic questions such as ‘What do you remember about the birth and after?’. While some responses were prompted by the interviewer, others were spontaneously given by the interviewees (see Table 1 for the interview guide). Interviews were recorded with consent and transcribed verbatim by YS, who also translated significant statements or ‘meaning units’ into English (Creswell and Poth, 2018, p. 79). The quality of the translation was validated by the second author, RS, who verified the fit of the significant statements with the identified codes, sub-themes and themes (Van Nes et al., 2010). When there were discrepancies, YS and RS discussed whether these were due to the lost nuances in the translation process or due to differences in the interpretation of the data. Basic demographic and background information was collected through a one-page questionnaire filled out by the respondents prior to the interviews. An average face-to-face interview lasted 78 min (ranging from 40 to 115 min) and were all carried out between May and June 2011. They took place at participants’ homes (*n* = 9) and in a park close to one of the participants’ office (*n* = 1). As the study was interested in the reflection of these women’s experiences, the women were interviewed one to maximum 5 years after their birth experiences in Austria.

**TABLE 1:** Interview guide and probe questions.

Q1. How did you end up giving birth in Austria?	
	What was your parents’ reaction? Did you consider going back to your home country to give birth? What kind of jobs did you have before and after birth?
Q2. What do you remember about the birth and after in the hospital?	
	Did you breastfeed and how did it go? What were you told to eat or drink, or not to when breastfeeding? What do you remember the most in the hospital? How satisfied were you with the food in the hospital? How satisfied were you with the amount of rest and sleep you got in the hospital?
Q3. Could you explain a typical day at home right after birth?	
	Was your husband able to take leave from work? Who provided support at home and what kind of support did you get? How satisfied were you with the food you had at home? How satisfied were you with the amount of rest and sleep you got at home? What did you do when you were not feeling well? Did you take any Chinese/Japanese medicine during the postnatal period?
Q4. How do you keep in contact with your family back home and your Chinese/Japanese friends in Austria? How often do you stay in touch?	
	Around the time when you were pregnant/after birth? Around the time after birth? Now?
Q5. Could you describe yours and your family language skills and inter-cultural experiences?	
	How would you rate your German, English or any other language skills? How well does your husband speak Chinese/Japanese/German? How well do your parents speak English or German? Have they ever lived outside their countries of birth? What language(s) do you use when you speak to your husband, parents and child(ren)? Does your husband also take Chinese or Japanese medicine?

YS, who is a Japanese woman married to an Austrian, has also given birth in Austria. She had not experienced postnatal acculturative stress. YS was highly aware and reflective of her position and how her own experience as a Japanese immigrant mother, as well as one who had not experienced PAS, could influence the interview and the analytical process. When women showed interest or asked for personal views, YS shared her own experiences and let the women react or comment on them. Thus the story telling and the analysis were done ‘together’ with the women during the interview process (Rapley, 2004, p. 22). One Japanese woman was acquainted with YS before the study commenced. All interviews were consensually audio-recorded with a digital device except for one, which was not fully recorded due to a defect in the device at that time point. The transcript in this case was re-constructed from field notes and follow-up email interviews. Information collected from seven follow-up telephone and email interviews, as well as memoranda, were added to the final data.

### Data Analysis

YS has a background in public health (MSc) with expertise and teaching experiences in qualitative methodologies. RS is a researcher in social medicine with training in qualitative research methodologies, and experience in psychosocial support for marginalized populations. MK is a professor with background in quantitative and qualitative psychology, medicine and epidemiology. First, YS made a biographical summary and notes of all participants to obtain a deeper understanding of each woman’s biographical history (see Supplementary Appendix A). Second, after familiarizing herself with each of their stories, YS identified all meaning units (short sentences selected from the interview transcripts) that were thought relevant to the research question. Women’s lived experiences during the postnatal period were explored through developing free codes following Larkin et al.’s (2006) phenomenological approach. For this process, YS consciously tried to ‘bracket out’ Berry’s (1997) acculturation model in order to concentrate on the phenomenon in concern (Creswell and Poth, 2018). Thirdly, following the procedures of directed content analysis (Hsieh and Shannon, 2005), Berry’s four acculturation strategies: assimilation, separation, integration and marginalization were applied in a deductive way as pre-fixed but flexible codes. Analysis focused on women’s cultural orientation (attitudes) as well as what women actually did (acculturation behavior). These pre-fixed codes were expanded or moderated when considered necessary. As a fourth step, YS developed a codebook, which was used by RS to code the translated version of the meaning units (see Supplementary Appendix C for the codebook). We ensured inter-coder reliability and the quality of translation based on the principal of ‘group consensus as an agreement goal’ and not on inter-coder agreement rates (Saldana, 2011, p. 28). This process was achieved by discussing each case of discrepancy and disagreement between the two coders. Finally, codes were grouped into subordinate and superordinate themes. Definitions of themes were discussed and agreed among all authors and the conceptual frameworks were finalized with the senior researcher MK who was not involved in the analysis of the data. Our intentions were to relate our interpretations with pre-existing knowledge and integrate our findings into a broader theoretical context (Smith et al., 2009). Thus we analyzed our data adhering to two types of hermeneutical circles: one that moves between the ‘part and the whole’ and the other one that moves between the ‘fore-understandings’ and new ways of understanding the ‘fore-understanding’ (LeVasseur, 2003; Smith, 2007, p. 5). Table 2 provides an example of the analytical process.

**TABLE 2:** Example of the analytical process using the codebook.

Meaning units	Codes	Subordinate themes	Domain	Cultural orientation and behavior*
Don’t you think it’s insane? I thought in Japan one has to rest and lie down for a month after a cesarean but I smiled and said ‘OK! I do my best!’ (Akiko from Japan)	1. Cultural practices not understood or respected; 4. Frustrated and irritated; 5. Unexpected and unbelievable; 6. Living up to the expectations of unexpected 38.1. Context: Hospital 39.2. Person: nurse	A. Surprised and irritated with the consequences of not being able to share health belief and practice with the host society and its people	40.1 Postnatal rest	41.3 Passive assimilation
Yeh [but] what can they do? They are so far away. They tell me and send me e-mail whatever the things I can try [to improve health and increase breast milk]. But it was difficult. Although my husband [was] was taking 2 weeks off vacation. But still he was trying to help to cook but it is still different from having a mother here or whomever relatives here (Lien from Beijing, at home).	4. Frustrated and irritated; 10. Postnatal support from people who share the same health beliefs is hard to replace 11. Feeling physical and emotional distance with the family back home 14. Diet and breastfeeding 38.2. Context: Home 39.12. Person: Family and friends back home	A. Surprised and irritated with the consequences of not being able to share health belief and practice with the host society and its people. B. A lonely and isolated struggle.	40.2 Postnatal diet 40.3 Social support	41.3 Passive assimilation
My husband and I tend to discuss thoroughly what we do not agree or understand and try to come to a conclusion but in regards to out disagreement on at what stage we can take a newborn out into fresh air, I thought as long as all the babies here are doing like that, it cannot be a bad thing. “When in Rome do as Rome does” [laugh]. If my child were 100% Japanese I might have insisted on the ‘Japanese way’ but she is 50% Austrian. So I thought it was not my place to say anything about it (Kanako from Japan).	26. Lacking agency/autonomy 22. I: Accepting the destiny of becoming a permanent immigrant in Austria; 39.13. Person: Family Austrian husband; 38.2. Context: Home	C. Becoming a mother and becoming a ‘permanent’ immigrant — acculturative stress in a context of a dual identity change	40.5 Identity	41.3 Passive assimilation
My mum did [all the cooking]. She was in charge of everything [big laugh]. My [Austrian] husband has a very good relationship with my parents and he understands that I moved my entire life over here for him. And so you know its fine having my family and friends over to stay (Grace, second generation Hong Kong Chinese).	32. Trusting and respectful relationship with the significant others 33. Trusting and respectful relationship between the significant others; 36. Personality: extroversion 39.13. Person: Family Austrian husband; 39.16. Person: Family mother; 38.2. Context: Home	D. Trust and mutual respect with and between the significant others	40.2 Postnatal diet 40.3 Social support	41.6 Integration

*Directed content analysis using Berry’s acculturation coping strategies as pre-fixed codes.

### Rigor and Reflection

To ensure rigor in analysis and reporting, we were guided by the ‘Consolidated Criteria for Reporting Qualitative Research (COREQ)’ (Tong et al., 2007). Our study fulfils the criteria listed in the 32-item checklist of COREQ (see Supplementary Appendix B). Transcribed interviews and preliminary findings were sent back to all participants for validation and comments. The data analysis software Atlas.ti was used to code, re-code, manage and re-organize the data. The researchers were open and reflective through the entire hermeneutic circle on their personal and professional backgrounds that could influence the interpretation of the data.

## Results

### Demographic Characteristics

Participants were all married, had more than a college education, and had health insurance. Their profile varied in terms of spouses’ nationalities, years living in Austria and self-rated German skills. Two Chinese-speaking participants had been living and working in Austria for more than 15 years, had a wide social network of Austrian friends, spoke German fluently and were thus highly acculturated to the Austrian society. One Japanese woman, Noriko, was intra-culturally married. Grace was the only second generation immigrant born in the United Kingdom (U.K.), thus highly acculturated to the West. Three women had an unplanned cesarean section. The demographic characteristics of the women are summarized in Table 3. A more detailed biographical summary of each woman can be found in Supplementary Appendix A.

**TABLE 3:** Demographic characteristics of the participants.

Pseudonym	From	Age at giving birth in Austria	Age at interview	Working status	Spouse from	Parity	C-section	Hospital	Years in Austria at the time of interview	German language 1- poor to 5 - fluent*
Lien	Beijing	43	45	Maternity leave	Austria	1	Yes	Private	15	4
Emily	Taiwan	36	41	Self-employed	Germany	1	No	Public	19	5
Jane	Beijing	41	45	Full-time working	Southern Europe	1	Yes	Private	8	1
Grace	U.K.	30	33	Maternity leave	Austria	2	No	Public	5	4
Akiko	Japan	34	38	Half-time	Austria	1	Yes	Public	5	4
Noriko	Japan	30	35	Not working	Japan	2	No	Public	8	3
Emi	Japan	29	33	Not working	Austria	2	No	Public	7	2
Ayako	Japan	30	33	Not working	Austria	2	No	Private	4	2
Maki	Japan	41	45	Not working	Austria	1	No	Public	8	3
Kanako	Japan	38	40	Not working	Austria	1	No	Public	2	1

*Self-rated.

### Findings From the Qualitative Analysis

All interviewees pursued some elements of *zuò yuè zi* or *satogaeri*. Based on our definition, we identified nine women experiencing PAS. Stress was identified in four specific domains: postnatal rest and diet (*n* = 9), social support (*n* = 7), feelings toward their significant other (*n* = 8) and identity (*n* = 6). Grace from Hong Kong was the only woman who did not articulate any experiences of PAS. Our findings are organized in three parts: (1) PAS in the lived postnatal experiences; (2) factors preventing or mitigating PAS; and (3) relevance and limitation of Berry’s acculturation model. The quotes presented here were selected based on the COREQ guidelines: namely, those that best illustrated the themes and findings of the study from a wide variety of participants (Tong et al., 2007).

#### PAS in the Lived Postnatal Experiences

Based on the qualitative phenomenological approach, we identified the PAS in the lived postnatal experiences as an ‘unexpected solitary struggle in the midst of dual identity change.’ This comprised three subordinate themes: surprised and irritated; lonely and isolated; and becoming a mother, as well as becoming a ‘permanent’ immigrant.

##### Surprised and irritated

This subordinate theme depicts the irritation, frustration and the feeling of unexpectedness for not being able to share their health beliefs and practices with the host society and its people. It also includes unexpected feelings toward their significant others. Lien, Emily, Jane, Akiko, Noriko, Emi, Maki, and Kanako all articulated that there were unanticipated expectations that surfaced through cultural differences and norms relating to postnatal maternal health. This was expressed more intensely by first-time mothers or through stories related to first birth in Austria. In the hospital, many respondents did not expect to be roomed-in with their infants immediately after birth. They felt unprepared to take care of their newborn while they themselves were supposed to be resting. Akiko thought that the nurses were *“crazy”* by asking her to stand up and start walking one day after the cesarean section.

“I thought ‘in Japan usually you have to rest and lie down for a month’ but I smiled and said ‘OK! I do my best!”’(Akiko from Japan).

Respondents felt their culture of postnatal rest was neither understood nor respected by the hospital personnel or their roommates. These negative emotions were sometimes compounded by the experience of perceived discrimination.

“*The mobile phone of the lady next to me was ringing all the time even after the visiting time was over and she was non-stop talking. From morning to night it was never quiet. I wanted to question the nurses who fiercely sent my husband away just because the visiting time was over. I really had problems with those nurses who changed their attitudes once they knew I could not speak German. That is against a nightingale’s mentality” (Kanako from Japan).*

For all ten women, diet was an important component of the postnatal recovery phase, and especially for producing good quality breast milk. They felt that the hospital food was insufficient in quantity, quality, and content. In addition, they found that it was neither suitable for their health nor for the production of breast milk.

“Once I had white fish with potatoes and I think that was the best thing that I ever got. The rest was like ‘really? Is this dinner?’ — a kind of cake with very sweet vanilla sauce. That was dinner [laugh]. I cannot possibility eat that. It was pretty bad” (Maki from Japan, in a public hospital).

Respondents who gave birth in private hospitals found the quality of food acceptable but not helpful in producing breast milk.

“I desperately wanted to feed my baby myself but it just did not work. [The hospital food] was really good but it is not the traditional way of the Chinese to drink a lot of [western] soup. They [the hospital] got good food but still I do not think that [kind of] food helps producing milk. In China — maybe like Chicken soup, or pork-feet food” (Lien from Beijing, in a private hospital).

Jane from Beijing was aware that she would not be satisfied with the hospital food. She prepared her *zuò yuè zi* meal in advance and had it brought in and served to her by a privately hired external midwife. Upon realizing that the hospital staff disapproved of external food being brought in for her, she felt guilty and could not enjoy her *zuò yuè zi* meal to full satisfaction. Nevertheless, she continued consuming her own food, as she strongly believed in its healing properties.

“They [the hospital personnel] did not allow me to have my own food. But you know for Chinese we have to have certain special drink to clean up. We call it “shçng huà tâng” (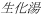) — to get rid of unnecessary [things] in the womb. So I asked my private midwife and she helped me to have it in the fridge and warm it up. Illegally, I had a little but not much” (Jane from Beijing, in a private hospital).

Many of the women discovered new sides to their husbands during the postnatal period. In the cases of Akiko, Emi and Kanako, these were expressed as negative surprises and disappointments to their Austrian husbands’ poor understanding to women’s heritage culture.

“*It was a big mistake not preparing my own food for the hospital stay. I should have packed my bag myself. My husband cannot even identify the [instant] miso-soup packages. I told my husband that there’s these kind of food in the cabinet at home so please bring them over and he brings the totally wrong things — like dried seaweeds” (Akiko from Japan).*

Negative surprise, however, was also expressed by an intra-nationally married woman. Noriko was disappointed with her Japanese husband for not being able to support her due to his poor German skills.

“So I said ‘wake up! wake!’ and woke my husband up and told him that the water has broken and we have to go to the hospital urgently. But he is totally panicking. So I called the ambulance myself and the ambulance came but my husband was totally useless. My husband speaks zero German so the ambulance crews were asking lots of questions to me [laugh]. Really no help at all. Not useful being around” (Noriko from Japan).

##### Lonely and isolated

The second subordinate theme illustrates the respondents’ feelings of loneliness, sadness, and lone struggle in dealing with their complex emotions and realities related to their cultural differences and health beliefs. Naturally, the feeling of being lonely and isolated was related to the lack of social support or not having family members from home with them during the postnatal period.

“They are so far away. They tell me and send me e-mail whatever the things I can try [to get the breastfeeding going]. But it was difficult although my [Austrian] husband was taking two weeks off vacation. But still ummm he was trying to help to cook but it is still different from having a mother here or whomever relatives here” (Lien from Beijing).

Nevertheless, it is important to point out that some of these women (Akiko, Emi and Kanako) experienced psychological loneliness as a result of their Austrian husband not having supported their health beliefs. These women struggled alone. Their sense of agency was felt to be undermined by the unequal power balance in favor of their husbands who upheld the ‘dominant’ cultural knowledge of their society. These Austrian husbands symbolized the patriarchal family structure, regarded as holding the general decision-making power in the family, not primarily due to gendered relations but because of husbands’ cultural dominant position in the Austrian society. Emi from Japan expressed appreciation for her husband in caring about her health. Nevertheless, she implied frustration due to her lack of power and agency.

“I had severe constipation after birth and it was so painful that I told my husband that I wanted to change my [fiber-rich insulin-level-control] diet [prescribed by the hospital] to something else. But he said ‘Fiber-rich food usually helps constipation.’ So I showed him some information from the Japanese websites that said excess fiber can also cause constipation. ‘See? It’s written here,’ I said. Then he tried to frighten me by saying ‘Do you want to be sick later? Then you won’t be able to eat anything!’. I pleaded and told him I wanted to quit this diet that made me grunt every time I went to the toilet. So I cheated a bit and when my husband was not watching, changed my diet a bit. I was not allowed to eat what I wanted to eat for one to two months. A pretty long time” (Emi from Japan).

Experiencing loneliness and isolation was compounded by the feelings of not being able to share complex emotions nor be supported by the most important people nearby. Feeling psychologically isolated was closely linked to the first subordinate theme of being surprised and disappointed with the respondents’ husbands. It also closely relates to the last theme on trustful and respectful interpersonal relationships with the significant other, which will be elaborated later.

##### Becoming a mother and becoming a permanent immigrant

The third subordinate theme illustrates identity change being an especially complex process for immigrant women after birth. By having a child in Austria, the women in our study became more psychologically ready for assimilating into the Austrian society and its culture. This was most strongly expressed by women married to Austrian men. Women’s assimilative behavior and attitude was partly a result of showing respect to their Austrian husbands. On the other hand, their own cultural identity became more evident as the desire to pass on their cultural heritage to their children grew. Lien, Emily, Akiko, Emi, Ayako, and Kanako all expressed ambivalent feelings toward becoming a mother and becoming a permanent immigrant to Austria at the same time. Until childbirth, these women typically felt that they were ‘temporary’ immigrants, having the option of going back to their home country or pursing their careers elsewhere. All six of them had discontinued or given up their careers before moving to Austria. After having a child in Austria, some of these women settled with the thought of making Austria their home and becoming ‘permanent’ immigrants.

“*My final wish was to go back to Taiwan for work. Now I do not persist [on] this point any more — [not] so strongly as before. Because if you have a child your presence is important. I can accept being here” (Emily, from Taiwan)*.

The identity change and accompanying acculturative behavior is influenced by the complex emotional feelings toward the respondents’ husbands. Pregnancy provided good reasons for the women to go back to their home country for a longer period of time but this meant being separated from their husbands for a long time. Ayako had decided to go back to Japan to do *satogaeri* for her first child but did not do the same for her second. She wanted to ‘respect’ her husband’s feelings and gave birth in Austria for the second child.

“[For the first birth], I thought satogaeri would become my last chance to go back to Japan for a long-term period. I wanted to do satogaeri for the second one too, but I sort of showed respect to my husband” (Ayako from Japan).

Women also started to accept that their child would become more ‘Austrian’ than themselves. Again the power balance and interpersonal relationships with the respondents’ husbands influenced women’s attitude and behavior.

“In regards to our disagreement [with my Austrian husband] on what stage we can take a newborn out into fresh air, I thought as long as all the babies here are doing like that, it cannot be a bad thing. “When in Rome do as a Roman does” [laugh]. If my child were 100% Japanese I might have insisted on the ‘Japanese way’ [of staying inside for at least a month] but she is 50% Austrian. So I thought it was not my place to say anything about it” (Kanako from Japan).

On the other hand, having a child made many of these women aware of their increasing need for support from the same-cultural group, and of their willingness to pass on their cultural heritage to their child.

“Because I realize how important that relative relationships are: grand-mum, grandparents my sister. This kind of relatives’ nets — ‘sozialnetz’ [social network] is very important after I have a daughter. Because when I was a student I did not have too many connections with Chinese students. So after I have a child, I get more in contact with Chinese mamas. But umm to be a real close friend you need time [at least] for me. And I just try to organize something to meet each other to practice language or to get a chance for speaking [Chinese]. Because I am the only person who speaks Chinese to my child except [when] she talks to her parents with Skype or telephone or something. So I think every chance is quite ummm how to say precious” (Emily from Taiwan who studied in Austria).

Childbirth also provided occasions in which the respondents came in intensive contact with the host country’s bureaucracy and healthcare personnel. These interpersonal experiences with the host population had influence on shaping women’s identity. Lien from Beijing, for example, encountered a series of bureaucratic hurdles with the city government right after birth in the process of getting her child’s Chinese name officially recognized and registered. *“Here [in Austria] I wanted to give my child a Chinese name.”* Lien was in fact the only woman interviewed who had obtained an Austrian citizenship and had given up her Chinese one several years before pregnancy. She was highly upset and surprised to know how difficult it was to give her child a Chinese name in Austria. Kanako’s negative experiences with the nurses in the hospital made her doubtful about her identity as an open, multicultural and international person. Further, recognizing the fact that she had transformed from a temporary into a permanent immigrant in Austria by having a child made her feel ambivalent about her situation.

“Basically I have a policy of enjoying my life wherever I go. Up until now I never really had problems with foreign languages or living in foreign countries so I feel like a failure here. I think I am not fated to be in this country” (Kanako from Japan).

#### Factors Preventing or Mitigating PAS: Trust, Mutual Respect and Interpersonal Relationships

From the inductive analysis, trust and mutual respect were identified as the last subordinate theme of respondents’ postnatal experiences (see Appendix C). We interpreted trust in self and others; trust and pride in own cultural tradition and health beliefs; and mutually respectful interpersonal relationships with and between the respondent’s significant others as important factors in preventing and mitigating PAS. Respect was expressed by positive feelings of appreciation and recognition. Trust was expressed in terms of confidence, belief and faith in oneself and others, as well pride in own cultural tradition. The lack of these elements exacerbated acculturative stress and led to negative emotions. Although this trend was identified among all ten women, the case of Grace, who had not experienced PAS, specifically illuminated the possible psychosocial pathways in which PAS could be prevented. Grace’s case was unique as she was highly westernized due to being a second-generation but she was not highly acculturated to Austrian culture. Grace revealed a very strong outgoing and optimistic personality but above all, she highly trusted herself as well as her heritage tradition and health beliefs inherited from her parents. Grace’s mother was a first-generation Hong Kong Chinese woman in the UK. She was someone Grace could look up to, respect and trust. Thus the trust in self as well as in her heritage culture enabled Grace to make selective choices related to what to take from her heritage culture and what from the Austrian health beliefs. Grace’s Austrian husband and the mother were both described as empowering persons who respected Grace’s choices and decisions. She especially expressed appreciation to her husband’s openness to her culture and his good relationships with her family. We interpret that these elements of trust in self and own tradition, and mutually respectful relationship with her husband and mother, as well as the positive relationships they had between them, prevented Grace from experiencing PAS in the first place. Relevant quotes from Grace are provided in the following part.

Respondents expressed varying degrees of trust in themselves and in their culture, as well as had different interpersonal relationships with their caretakers and significant others. Three Chinese-speaking respondents (Lien, Jane, and Grace) explicitly expressed the feeling of pride in their medical tradition and appreciated that the host population recognized it.

“Then I also saw some Austrian customers [in the Chinese medicine shop in Vienna] — not Chinese — and it seems they also do something [with Chinese medicine]. Yes it’s a progress. 10 years ago, when I first came here it was not really like, people did not believe in that. I guess they do not find the answer from West[ern] medicine. But in China I think the medicine [is applied in] two-ways, Western and China combined. It has been there [like that for] many years so actually I think that is good. I guess you need both”(Lien from Beijing).

Pride in their own health beliefs helped these women to select or apply different medical beliefs in a complementary way and prevent PAS.

*“I think she [the midwife] told me certain knowledge [related to what is good for producing milk]. But I do this with the pig [feet] soup? With peanuts and ginger and I did that and it works” (Jane from Beijing)*.

Another important source of confidence was the support from their non-Chinese husbands in their health beliefs. Lien, Jane and Grace all expressed appreciation and respect toward their husbands.

“I think my husband he accepts it [Chinese medicine] although he said it’s really horrible to drink. He really accepts it. He would take it because there’s [real positive] results. He believes in it” (Lien from Beijing).

In contrast, lacking trust in their own medical beliefs and the absence of a mutually respectful relationship with their husband led some of these women to experience very high levels of PAS.

“I do not know why but here they say the entire opposite of Japan. In Japan they say one should eat lots of root vegetables with fiber. Beans are also good. Eat those kinds of things, lots of vegetable and produce milk like a cow, don’t they? When the baby cried my diet was blamed. Starting with my husband as the closest enemy and naturally my mother-in-law. Even the doctor. Everyone said like that. Can you believe it?” (Akiko from Japan).

Mutually respectful relationships between women and their mothers were identified as further important factors in mitigating PAS. Emily, Grace, Akiko, Ayako, and Kanako had their mothers coming over in the postnatal period to help them out with the *zuò yuè zi*, or practiced the reversed *satogaeri* custom. Noriko received support from her mother-in-law as her own mother was too ill to fly over. While these women appreciated their mother’s or mother-in-law’s willingness to support, they also expressed ambivalent feelings toward their mothers and the cultural practices of support. Akiko, for example started to doubt the benefits of the postnatal support.

“If my mother hadn’t come to help and I had to cook myself, I would have had no choice but to just leave the baby crying. So I would not have had to devote all my energy for preventing the baby from crying. Cooking would have been nice for a change. It was a torture that the only thing I was allowed to do was to take care of the child” (Akiko from Japan).

Respondents also realized that their parents’ acculturation and understanding of the Austrian context was important. The parents’ lack of acculturation to the Austrian culture intensified respondents’ acculturative stress.

*“My parents are not so traditional but my mother was not satisfied after [my] pregnancy. Actually my mum came here and I was cold because Taiwanese people are not used to cold. It was January. My mother did not find the things so convenient here as she wished. She also brought some Chinese medicine. But how to say, the condition [here] is quite*…*totally different” [from Taiwan] (Emily from Taiwan).*

On the contrary, Grace from Hong Kong, who did not experience PAS, expressed a mutually respectful relationship with her mother which enabled her to make selective choices.

“Things like cauliflower and broccoli she [my mother] would not have thought about [that they would cause colic]. I told her not to [cook them] because [I have heard that] it would cause wind in the baby and she listened to me as well [and did not cook].”

Interpersonal relationships between these women’s mothers and husbands were also critical factors in preventing or exacerbating acculturative stress.

“*My mother was highly frustrated with my [Austrian] husband being a vegetarian. You know hardcore Japanese like her are unforgiving toward vegetarians. She completely lost her temper when my husband refused to eat because my mother accidently poured a bit of fish broth soy sauce over the food” (Ayako from Japan).*

In contrast, Grace stated:

“My [Austrian] husband has a very good relationship with my parents and he understands that I moved my entire life over here for him. And so you know its fine having my family and friends over to stay” (Grace, second generation Hong Kong Chinese).

#### Relevance and Limitations of Berry’ Acculturation Model

**FIGURE 1:**
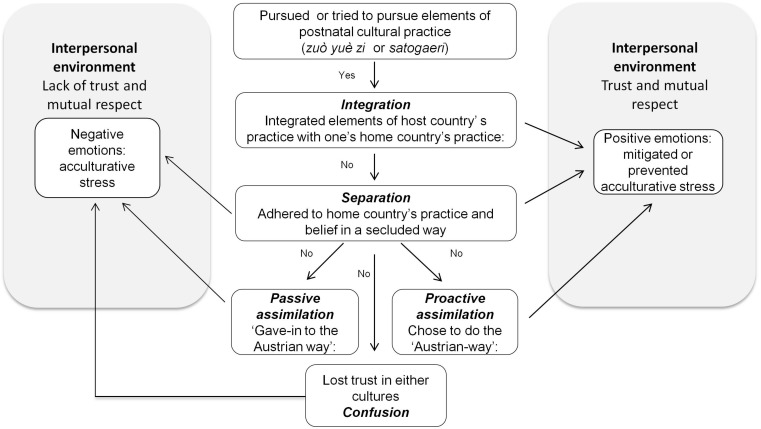
Conceptual framework of acculturation behavior and postnatal experience.

Our deductive analysis revealed that the postnatal behavior or acculturative experiences described by the women could be grouped into Berry’s (1997) pre-established categories of integration, separation, and assimilation. Marginalization was not identified as a ‘behavioral strategy’ but a similar notion of ‘confusion’ — losing trust in either of the cultures — emerged as a categorical concept. We also identified two types of assimilation patterns — proactive and passive. Women did not describe one type of acculturation pattern but multiple ones depending on the domain and the context (for example, hospital versus home). Three acculturation patterns: separation, integration and proactive assimilation were all related to the positive emotions of trust and mutual respect, and contributed to preventing or mitigating PAS. At the same time, separation, without the elements of trust and respect was related to negative emotions. Confusion and passive assimilation were expressed together with the feeling of lacking trust and mutual respect, and led to the exacerbation of PAS. The conceptual framework is depicted in Figure 1.

Only Grace from Hong Kong and Maki from Japan described what we could label as *integration*. Grace shared her postnatal *zuò yuè zi* meal with her Austrian husband while Maki integrated both Austrian and Japanese ingredients into her postnatal diet. They both expressed trusting and respectful relationships with their caretakers. Maki’s experience with the home-visiting midwife also illustrates the case that having a trusting and mutually respectful relationship with the caretakers mitigates stress, and is an important positive factor that enables integration. Maki, who had severe postnatal hemorrhage, did not get any help from her family. She experienced acculturative stress in the hospital and loneliness at home. However, being in charge of her diet at home by integrating her Japanese diet with what she learned from the trusted midwife gave her a feeling of empowerment, and helped in alleviating her stress.

[When I was breastfeeding] I had lots of cereals, seaweeds and vegetables. I like vegetables very much. The home-visiting midwife [who voluntarily visited me] taught me this Austrian recipe — a fried celery — telling me it will give me an energy boost so I cooked that and ate it too (Maki from Japan).

*Separation* was described by Ayako, Noriko, Grace, Jane and Lien. Other than Noriko, all of them made it explicit that they consciously separated their practice from that of the Austrians. Noriko’s separation behavior was rather unintentional as Noriko’s husband and mother-in-law were both Japanese. This created a natural environment in which Noriko could pursue the *satogaeri* practice privately. She trusted her mother-in-law and appreciated her support. Jane from Beijing consciously secluded herself to her Chinese friends, whom she could trust to support her in her practice of *zuò yuè zi*. She deliberately excluded her Western husband from this process.

He [my European husband] got a one month paternity leave but he did not take it immediately after birth because you know the baby does not really need papa [for the zuò yuè zi] so he kept it for later. During the month I had my friends help me [to do the zuò yuè zi]. These friends were already engaged. Three Taiwanese, one Malaysian but all Chinese origin (Jane from Beijing).

Noriko and Jane both expressed positive postnatal experiences at home. Ayako also tried to pursue the modified *satogaeri* with her mother who came over to Austria. However, this attempted separation led to additional stress as she was caught between the intercultural conflict between her mother and her Austrian husband as illustrated in section “Factors Preventing or Mitigating PAS: Trust, Mutual Respect and Interpersonal Relationships.”

Akiko, Emi, Noriko, Emily, Grace, and Lien all described behaviors that we coded as *proactive assimilation*. This included actively learning the language (in contrast to passively learning the language), learning from experiences and consciously replacing their cultural practice with the ‘way things done in Austria.’ Naturally, some of the women who had prior experiences giving birth in Austria were able to trust oneself in the subsequent situation better, felt more comfortable with the experience, and hence “proactively” assimilate.

“*In the end, I was happy that I did it the ‘Austrian way.’ It’s your own child and it was taken for granted [in the hospital] that you take care of your own baby. My Japanese friend who gave birth in Japan told me ‘it was so comfortable and relaxing being in the hospital for a week!’ I could not have had my own baby taken care for by another person” (Emi from Japan, second birth).*

Not only building trust in oneself but trusting the Austrian caretaker and the system made the proactive assimilation a satisfying experience. While Grace from Hong Kong shared her *zuò yuè zi* meal with her Austrian husband (*integration*), when it came to choosing between Chinese and Austrian health beliefs with regards to breast milk, Grace decided to trust her Austrian nurse and followed the ‘Austrian way.’ Again, the mutually respectful relationship with her supportive mother empowered Grace to select and assimilate in the domain of her choice.

On the other hand, *passive assimilation*, articulated by Akiko, Emi, Noriko, Jane, and Lien, was predominantly expressed as negative emotions and submissive reactions to acculturative stress. Although these women were inclined to more passively assimilate within the hospital setting, the stress experienced at home was more intense. As described in section “PAS in the Lived Postnatal Experiences,” Emi from Japan lacked a mutually respectful relationship with her husband and accepted the Austrian diet that was strongly recommended by him. She was highly dissatisfied with her diet for several months after birth. Similarly, Akiko from Japan felt that she could not trust anyone with regards to the maternal diet and was highly discontent with her choice of integrating the Austrian practice.

Akiko and Kanako from Japan and Emily from Taiwan articulated a sense of *confusion* — losing trust and cultural orientation to either of the cultures. Despite considering themselves as ‘bicultural,’ ‘international’ and being highly acculturated to Western values and languages, both Emily (Taiwan) and Kanako (Japan), expressed this sense of being lost and confused. Akiko, on the other hand, lost her trust in both cultures as far as her maternal nutrition was concerned, and expressed the highest distress among all respondents. She diagnosed herself as having an ‘obsessive–compulsive disorder’ in trying to follow the ‘correct’ diet for breast milk production. Many other of these women expressed similar feelings of confusion and disappointment with their husbands (Akiko, Noriko, Emi the Kanako), ambivalent feelings toward their mothers (Akiko and Emily) and unforeseen conflicts between their husbands and mothers (Ayako). These feelings of confusion were one of the causes, and exacerbating factors of PAS.

Finally, it is important to point out that each of these women articulated multiple ways in dealing with, mitigating or preventing PAS. Women who assimilated in the hospital resorted to separation or integration at home. Many also assimilated within the domain of postnatal rest but continued separation with their postnatal diet. By having a child, women became mentally prepared to assimilate into the Austrian society as a permanent immigrant but at the same time, strengthened their non-Austrian identity (separation). Acculturation behaviors were also not always a result of conscious strategy. For example, passive assimilation was often a result of having no choice. Integration and confusion were unintentional outcomes of the types of interpersonal relationships with and between their significant others.

In short, Berry’s acculturation model serves as a useful framework in analyzing acculturative behavior and maternal psychological health in the postnatal period. Nevertheless, the multifarious complexity involved in the process of acculturation and the experiences of acculturative stress makes it difficult to draw causal relationships between certain acculturation behaviors, strategies, or cultural orientations with specific health outcomes.

## Discussion

This paper explored the postnatal experiences of Chinese-speaking and Japanese women in the context of Austria. Our study is the first of its kind in investigating the interrelationships between acculturation behavior or cultural orientations, and maternal psychological health through the application of Berry’s model in a qualitative way. Many of our findings were in line with qualitative studies exploring the experiences of East Asian women adhering to postnatal practices in other Western countries. Similar to the women in our study, the negative emotions of helplessness and loneliness were identified among Chinese women interviewed in Australia (Chu, 2005). The specific context in Austria in which Asians are one of the extreme minority ethnic populations could have made the feeling of isolation worse. Our study also indicates a general lack of understanding to different health beliefs and limited cultural sensitivity in Austrian maternal healthcare setting. While some respondents expressed positive experiences with home-visiting midwives, many of them articulated negative experiences related to the attitudes and behaviors of the nurses, physicians as well as hospital support staff.

As suggested by Matthey et al. (2002); Grigoriadis et al. (2009), and Fung and Dennis (2010) the social support mobilized through the postnatal cultural practices prevented the women in our study from becoming physically isolated during their most vulnerable period after giving birth. On the other hand, the benefits of the cultural practices to the psychological health of the immigrant women is complex, given that the adherence to the practices could lead to both acculturative and enculturative stress as depicted in our study.

The central organizing concept of the ‘unexpected solitary struggle in the midst of dual identity change’ symbolizes the complex psychology of immigrant mothers who struggle to live up to multiple cultural expectations while transforming from ‘temporary immigrants’ into ‘permanent immigrant mothers.’ Similar types of struggles in meeting different cultural expectations are reported in a study among Korean women in New Zealand (DeSouza, 2014) and Chinese women in Scotland (Cheung, 2002a, b). The identity change illustrated in our study also resembles Weinreich’s (2009) interpretation of ‘enculturation’ as an immigrant’s ‘agentic’ process of revisiting and reconstructing one’s identity. Our study revealed that this process was experienced in a particularly intensive manner due to the immigrant woman having a child in a cultural context distant from her own. The postnatal period was experienced as a solitary struggle not because these women did not have any social support but because they came to recognize that they lacked orientation within themselves as well as in their culture, and they lacked the people with whom they could sincerely trust, respect and share their cultural beliefs.

In our current study, we also explained the psychological function of trust and respect — especially in relation to the interpersonal relationships with the spouses and caretakers. With regards to respect, one quantitative study reports ‘respect’ as being a protective factor against perinatal depression among Mexican immigrant women in the U.S. (D’Anna-Hernandez et al., 2015). Another quantitative study with adolescent Mexican mothers found that a perceived positive relationship with their own mothers lessened prenatal depression symptoms among the second-generation Mexicans but not among those from the first-generation (Zeider et al., 2015). Our study provides some insights into these complex psychological pathways between respect, trust and maternal psychological health and possible differences between the first and the second generations. The component of trust in oneself as mitigating factor against acculturative stress is illustrated by some qualitative studies in the past. The Chinese women in Scotland for example, ‘reconstructed’ their rituals by making an effort to ‘fit in’ while at the same time maintaining some elements of their cultural practices that they trusted (Cheung, 2002a, b). Inter-culturally married Japanese women in Hawaii developed confidence and trust in themselves from their previous birthing experiences in the U.S. and mobilized their own resources to counteract the lack of social support (Taniguchi and Baruffi, 2007). Within our sample of highly educated and financially stable women, the Chinese women expressed slightly more trust and confidence in their food and health beliefs than the Japanese women. One of the reasons could be that the protein-rich food consumed during *zuò yuè zi* did not contradict with the Austrian food beliefs while the fiber-rich diet based on vegetables and fruits preferred by the Japanese women did. The other reason could be related to the high trust and pride held by the overseas’ Chinese in their own medical tradition and history (Gervais and Jovchelovitch, 1998; Green et al., 2006; Kong and Hsieh, 2012) which is assumed not to be the case with the Japanese (Lock, 1980, 1985). Our study also implies some variations in the experiences of PAS between women receiving public and private care in Austria. Respondents who gave birth in private hospitals were generally satisfied with the quality of food but not with its appropriateness to maternal health while women who gave birth in public hospitals were highly dissatisfied with hospital foods in terms of quality, quantity and appropriateness. The possibility of communicating in English appears to be easier in private hospitals which reduced PAS related to communication among some of the respondents. Hiring private midwives, on the other hand, did not necessary lead to easing PAS related to diet in our sample and two women specifically expressed positive experiences with home-visiting public midwives. Although these aspects are beyond the scope of our current paper, future acculturation studies could explore the historical roots of pride in health-related cultural practices among immigrants and how this could influence their identity and maternal psychological health. Future studies could also investigate how maternal care delivered by the public or private sector could differ in regards to immigrants’ experiences of acculturative stress.

Finally, Berry’s (1997; 2005) model of the four “acculturation strategies” offered a useful framework for understanding types of immigrant women’s acculturative behaviors and how they could be related to maternal psychological health. Concurring with past findings (Berry and Sabatier, 2010; Gui et al., 2012), *passive assimilation* and *confusion* (or *marginalization*) were related to negative emotions while integration and separation were related to positive ones. In agreement with Berry’s (1997, 2006), an open and inclusive host society mattered to make the integration of immigrant women possible. Nevertheless, our qualitative analysis also showed that care should be taken in drawing causal links between acculturative behaviors or cultural orientations and health outcomes. Intermediate psychosocial and interpersonal factors such as trust and respect, as well as power dynamics in the family appear to have had major influences on the self-perceived health of these immigrant women. As shown in our study, separation and assimilation were related to both negative and positive emotions with intermediate factors playing important roles. Our study also reveals the complexity involved in determining immigrants’ cultural orientations and how these could be related to maternal psychological health. Those who declared themselves as bi-cultural experienced acculturative stress as much as those who considered themselves rather ‘mono-cultural.’ Objective measurements of acculturation, such as years of residence or language skills, did not indicate a clear-cut relationship with positive or negative emotions during the postnatal period. Our qualitative study also showed that acculturative behaviors are often unintentional and not all behaviors can be referred to as ‘strategies.’ Apart from those behaviors identified as proactive assimilation and deliberate separation, the acculturation behaviors of the women in our study were the outcomes of interpersonal relationships within specific ‘ecological’ contexts (Ward and Geeraert, 2016, p. 98). In agreement with Chirkov (2009a, p. 96), the word ‘strategy’ may portray the misleading assumption that immigrants can freely choose from different acculturation strategies. This could result in immigrants being blamed for choosing the ‘wrong’ strategy that affects their health.

### Strengths and Limitations

Our qualitative study is unique in a way that we combined an inductive and deductive approach in our hermeneutic circle. Given the large amount of associated knowledge and theories produced in the field of acculturation psychology, we were aware that we would not be able to completely ‘bracket out’ our preconceptions and professional experiences related to the topic. Instead, we openly attempted to ‘bracket in’ and ‘out’ our preconceptions in our analytical process (LeVasseur, 2003). This approach enabled us to appraise existing theories in light of our new findings (Smith et al., 2009).

A potential limitation of this study is the small sample size and the limited diversity in participants’ profile. In particular, the Chinese-speaking participants in this study were restricted to highly educated Chinese women from the northern parts of China and the findings may not be directly applicable to the majority of the Chinese population in Vienna who come from the southern regions of China (Zhao, 2010). Nevertheless, this sample frame enabled us to make comparisons between and among the participants and the two sub-groups. One of the strengths of our study was that the sample frame allowed us to discern cultural psychological factors that are not confounded by socio-economic factors. The sample size was small but considered sufficient to answer our research question (Patton, 1990). Moreover, our study illuminated the experiences of inter-culturally married women, which are rather neglected in acculturation research. Our goal was not to generalize but to expand our understanding to existing theories that can better inform practices aimed at responding to the diverse maternal health needs of minority immigrant populations.

Other potential limitations include recall and language biases. The upper-time frame of 5 years after birth may have resulted in recall biases among some of the participants. On the other hand, this timeframe allowed us to explore how these women reflected upon and made sense of their experiences after a certain time period had passed rather than trying to reconstruct what exactly ‘happened.’ We found that those interviewed 5 years after birth could vividly describe their emotions during the time after birth as much as those interviewed a year later. In addition, interviewing mothers with very small children were logistically and ethically difficult as it posed too much stress on the women. The two Chinese-speaking women who declined the interview both had small infants and time constraints. Seven women were interviewed in their mother-tongue (Japanese and English) while three Chinese-speaking women (Lien, Emily, and Jane) were interviewed in their second language (English). The use of foreign languages may have caused women to become more distant to their experiences and emotions (‘foreign language effect’) (Keysar et al., 2012). Moreover, foreign language is known to reduce mental imagery and may have hence influenced their memories (Hayakawa and Keysar, 2018). The use of foreign languages is also known to influence how honestly people talk (Bereby-Meyer et al., 2018). Being reflective of these biases, the interviewer encouraged the participants to freely use Chinese, Japanese, German, or English words whenever they felt these were more appropriate or better described their memories and emotions. Unclear meanings were clarified during the interview as well as through follow-up interviews.

The positionality of YS as a Japanese mother could have influenced the data collection and interpretation process. Nevertheless, sharing common cultural heritage and the interviewer’s understanding to intercultural and intergenerational complexity as an East Asian immigrant in Austria have likely made the participants trust and be open with the interviewer. Moreover, the intersubjectivity of both coders was reflected upon in all stages of the analytical process.

We used a codebook to ensure inter-coder reliability. We did not calculate the reliability rate as we checked our discrepancies each time after coding small parts of the transcripts (O’Conner and Joffe, 2020). We strictly adhered to the COREQ checklist by quantifying the results whenever appropriate, comparing findings with previous studies, and also reporting on deviant cases (Tong et al., 2007). Phenomenological study usually restricts study participants to those who have experienced the phenomenon of interest (Creswell and Poth, 2018). We included a participant who did not experience acculturative stress but could have had potentially experienced it. Although this is an unusual procedure, the inclusion of the exceptional case further enriched our understanding of the phenomenon.

## Conclusion and Implications for Practice

The postnatal period is a vulnerable time not only for immigrant women but indeed for all women (Razurel et al., 2011; Coates et al., 2014). Supporting new mothers to mitigate postnatal stress is one of the most essential components of postnatal maternal care (Fahey and Shenassa, 2013). Health professionals should be aware that immigrant mothers face particular types of stress related to acculturation. They go through a unique process of identity change in their postnatal phase that is not experienced by non-immigrant women. Health professionals could support immigrant mothers in mitigating PAS by accepting, respecting and not denying their health beliefs and practices as long as they do not pose obvious threats to maternal and infant health. They should be reflective of their own health belief systems and try not to impose them on the women. Immigrant women’s experiences with health personnel in the postnatal period influence how these women later perceive and integrate themselves into their host society. Nurses and home-visiting midwives could pay particular attention to family dynamics (for example, inter- or intra-national marriage, presence of parents and in-laws, power relationships within the family) and be aware of the fact that interpersonal relationships with and between the women’s significant others have a major impact on their postnatal acculturative experiences. They should be careful not to leave immigrant women alone with their own health beliefs. Acculturation theories and concepts should be included in the training modules of nursing practices related to maternal health. These training modules however, should focus less on the predictive value of the theories — those aspects that assume ‘culture’ or certain cultural orientations leading to certain health outcomes. They should recognize that a combination of personality, family, social environment and women’s migration histories have an impact on shaping immigrant women’s postnatal experiences and their health. Achieving this level of awareness among healthcare providers could promote access to and the provision of a more empathetic and women-oriented maternal care for women from diverse ethnic and cultural backgrounds.

## Data Availability Statement

The data supporting the findings of this study are available in the article and the Supplementary Material.

## Ethics Statement

The studies involving human participants were reviewed and approved by London School of Hygiene and Tropical Medicine, Research Ethics Committee. The patients/participants provided their written informed consent to participate in this study. Written informed consent was obtained from the individual(s) for the publication of any potentially identifiable images or data included in this manuscript.

## Author Contributions

YS designed the study, conducted the interviews, translated the interviews, conducted initial coding and primary analysis, and drafted the manuscript. RS-R verified the quality of the translation and ensured the validity of the codes, themes and the findings, and commented on the draft manuscript. MK supervised the study, provided input, and commented on the draft manuscript. YS, RS-R, and MK approved the final manuscript.

## Conflict of Interest

The authors declare that the research was conducted in the absence of any commercial or financial relationships that could be construed as a potential conflict of interest.
