# Supplementary material for: ‘No Austrian Mother Does This to Sleep Without a Baby!’ Postnatal Acculturative Stress and ‘Doing the Month’ Among East Asian Women in Austria: Revisiting Acculturation Theories From a Qualitative Perspective
Source: Front Psychol. 2020 May 14;11:977. doi: 10.3389/fpsyg.2020.00977 (PMC7240129; doi:10.3389/fpsyg.2020.00977)
Supplement: Supplementary file 1 [file Data_Sheet_1.pdf]

SUPPLEMENTARY MATERIALS

Appendix A. Biographical summary of each participant

Lien Lien was interviewed 2 years after her birth experience in Austria. A single child, she had already lived in Austria for 13 years before having given birth. She gave birth in a private hospital and had an unplanned cesarean section which was an unexpected and quite distressing experience that also influenced her postnatal mood. Lien has a college degree. Born and raised in Beijing, she met her Austrian husband through work in China and immigrated to Austria. She usually works full-time but was only working half-time to take care of her child at the time of the interview. She has spent almost one-third of her life in Austria (15 years). Her mother tongue is Mandarin Chinese but she speaks fluent German and English. After living in Austria for so many years, she thinks it is 'logical' to localize. Her Chinese identity became stronger in Austria but to avoid administrative problems she changed her nationality to Austrian. She was not the only participant who changed her nationality. She sees it as a "positive progress" that Chinese medicine has become accepted in Austria. Her pride and trust in Chinese medicine is high, and she still uses it complementary to Western medicine. She proactively took part in the publicly organized mother and baby meeting and interacted with other Austrian mothers. She did not have any Chinese family and friends coming to support her during her postnatal period. Her mother was too old to fly. She was slightly distressed due to her lack of family support. In general, she assimilated in the hospital and pursued the separation strategy at home. She believed in her Chinese diet with regards to what is good for breastfeeding and was not confused by Austrian beliefs.

Emily Emily was interviewed 5 years after her birth experience in Austria. A single child, she experienced a natural vaginal birth in a public hospital. Emily had prepared herself for natural birth through yoga and was quite proud of this. Although she was born in Taiwan and grew up in Taipei, she studied in Vienna and spent almost half her life in Austria (=19 years). She had lived in Austria for 14 years before giving birth. Her Austrian husband lived and worked in Taipei for 6 months and speaks Mandarin Chinese. She travels between Taipei and Vienna quite often and declares herself to be a 'bi-cultural' person. Emily has written a book comparing the Kindergarten and small child education between Austria and Taiwan. She has finished her PhD at an Austrian University and works as a freelancer. She had the highest education level among all women interviewed. She had planned her career back in Taiwan but after having a child, she more or less decided to settle in Austria. She has not, and does not plan to take up Austrian nationality. Her mother tongue is Mandarin Chinese and speaks fluent German and English. She did not experience much acculturative stress in the hospital as she had had prior knowledge on how the health system works in Austria. At home, she experienced some stress with her mother who flew over to provide support. Although she has many Austrian friends, she only came to realize the needs of Chinese-speaking friends after giving birth.

Jane Jane was interviewed 4 years after her birth experience in Austria. A single child, she had lived in Austria for four years before having given birth. She had an unplanned caesarean section in a private hospital. She was trying to avoid cesarean section, so this experience distressed her. Born in Beijing, she had spent almost half her life outside China due to study and work. Jane holds a postgraduate degree. She was back to her full-time job at the time of the interview. She works in an international environment where she mainly uses English. Her mother tongue is Mandarin Chinese and she speaks fluent English but very little German.. She moved to Vienna due to her long-term job. She had already lived in Vienna for 8 years but was not yet sure yet if she was going to permanently live in Austria. Jane's husband is a non-Austrian non-German speaking southern European. Jane speaks English with her husband. Jane's nationality is Chinese. She is very proud and confident about Chinese medicine but she wants her child to grow up as an independent person who is not spoiled by the parents and grandparents (like in China). Jane had no family support but proactively mobilized external resources such as hiring a private midwife in the hospital and organizing Chinese-speaking friends to help her pursue *zuò yuè zi* at home (separation). She pursued her practice not out of parental or cultural expectations but purely because she believed in it. She experienced acculturative stress in the hospital but not at home where she had control.

Grace Grace was interviewed three years after her first birth and one year after her second birth in Austria. She had spent less than two years in Austria before giving birth. Both children were born without caesarean but Grace had severe post-birth hemorrhage after both births and needed to be operated. This restricted her movements after birth but her husband provided most of the physical support needed. Her second child was born with a heart problem and had to be transferred to another hospital for an emergency operation right after birth. Around the same time, her mother-in-law was diagnosed with cancer and was hospitalized. Despite all these difficulties, Grace was the most positive and confident person among all and did not experience acculturative stress. Grace's parents were both born in Hong-Kong and moved to the United Kingdom (U.K.) where Grace was born. Grace spent most of her life in the U.K., where she completed her bachelor's degree and worked. She immigrated to Austria due to her Austrian husband's job. Grace's mother-tongue is English but she speaks Cantonese with her parents. She cannot read and write Chinese. She speaks English with her children but would like them to speak more Chinese. She speaks German quite well. Her husband had never been to Hong Kong but has travelled around the world. Grace praised her husband a lot in the interview for being very open to other cultures including the Chinese culture and its food. Grace highly expressed her trust and respect in her mother. She followed both her mother's and her Austrian midwife's advice with regards to the maternal diet and made conscious decisions as to whose advice to follow and when. The integration strategy was made possible due to the open and trusting relationship she had with her mother and her husband.

Akiko Akiko was interviewed four years after giving birth in Austria. She had lived in Austria about a year before giving birth. She had an emergency caesarean section at a public hospital which she described as a traumatic experience. Born and raised in Japan, Akiko spent almost all her life in Japan. Her mother tongue is Japanese and she speaks good English and German. Her confidence in speaking German was poor at the time of giving birth. Akiko has a postgraduate degree and had a prestigious job in Japan. Her relationship with her husband was complex as she felt she had sacrificed at lot for her husband by immigrating to Austria. She was the only Japanese woman among all Japanese participants that had a job in Austria at the time of the interview. She had no previous experiences living abroad but her father had been posted to the United States and her mother, who is an English teacher, had temporarily accompanied him. Akiko expressed the strongest feeling of distress during her postnatal period among all participants. She did not seek professional psychological support but diagnosed herself as suffering from severe postnatal depression. She experienced high acculturative stress with regards to maternal food beliefs and was confused in terms of what and who to believe.

Noriko Noriko was interviewed five years after the first and two years after the second birth in Austria. She had lived in Austria about three years before giving birth. She had normal births in a public hospital. Noriko is the only intra-culturally married woman. She moved to Austria due to her husband's job. Although it is not certain, she thinks the couple might stay in Austria permanently. Since her own mother was sick, her mother-in-law came to support her after birth, which she very much appreciated. She has a Taiwanese female friend who is her neighbor, and who also supported her after birth (not interviewed in this study). She had the least cultural conflict with her family members as they were all Japanese but she was slightly disappointed with her husband who could not help her due to his limited German skills. She was the only Japanese woman who did not express a need for social support after giving birth at home. She feels freer in Austria than being in Japan as she finds the fewer rules and Austrian way of doing things more relaxed.. She very much adapted and acted proactively in her second birth. She has a university degree. Noriko has never worked in Austria and at the time of the interview had no plan to work in Austria.

Emi Emi was interviewed seven years after the first birth and four years after her second birth in Austria. She had her first child in Western Austria far from Vienna. Although many years have passed, she could vividly talk about the acculturative stressful experiences she encountered during her first birth. She spent less than a year before giving birth to her first child in Austria. Emi met her Austrian husband in another European country where she was studying and planning to pursue her career in the future. She has a specialized college degree from Japan. Similar to Akiko, she felt she had sacrificed herself for her husband by moving to Austria. The

couple moved closer to Vienna when the first child was around two years old. Since she was not living in Vienna, she was socially isolated and was quite desperate to meet other Japanese mothers. Similar to Akiko, Emi experienced intense acculturative stress related to the maternal diet and felt isolated without being able to gain support from her husband. Nevertheless, she expressed confidence in going the 'Austrian way' with her second child. She has a strong desire to work in Austria in her profession but has been struggling with raising two children and learning the language.

Ayako Ayako was interviewed four years after her first birth in Japan and one year after her second birth in Austria. Ayako was the only woman who had experienced giving birth in her own country. She had studied and worked for more than five years in another European country before coming to Austria. Since she had never lived in Austria when she got pregnant, she decided to give birth in Japan for her first child. She thought it would be her last chance to stay in Japan for a long-term period. She very much enjoyed her pregnancy and birth (a real *satogaeri*) experience in Japan. For the second child, she decided to give birth in Austria out of respect and consideration to her Austrian husband. Ayako did not express much acculturative stress in the hospital as she had purposely chosen a private hospital where she could communicate with the health professionals in English. She was not surprised to be roomed-in with the infant immediately after birth because that was her experience in Japan too. She also did not expect much 'good' food in the hospital. Nevertheless, she experienced acculturative stress at home due to cultural conflicts between her Japanese mother and her Austrian husband. Although she appeared to be a well-prepared and confident person, this conflict seemed unforeseen to her. She has a university degree from Japan and was planning to work in her profession in Austria.

Maki Maki was interviewed four years after giving birth in Austria. She has one child — vaginal birth in a public hospital. She lived in Austria five years before giving birth. She used to privately work at home but stated she is a 'house wife' now without a strong intention to work. Her Austrian husband has mild disability and she did not want her parents to come to Austria so she was quite isolated after birth. She has one very close Japanese friend who she meets almost every day but other than that had very limited social network. Maki had severe bleeding after being discharged from the hospital and could not leave home. Maki has developed a trustful relationship with a public midwife before birth. This midwife came to voluntarily visit her at home after birth. Maki very much appreciated this support though she said her German language skill was very limited. She experienced acculturative stress in the hospital but at home could pursue integration due to this midwife. She has a specialized college degree from Japan.

Kanako Kanako was interviewed less than two years after giving birth in Austria. She has one child — having given a vaginal birth in a public hospital. She had only spent several months in Austria before she became pregnant. Similar to Emily from Taiwan, Kanako had many international experiences through her international profession which she left due to coming to Austria for her Austrian husband. She considered herself to be very international and multicultural and therefore was shocked to encounter acculturative stress in Austria. She considered her husband to be a very open and understanding person, and did not experience stress or conflict between her husband and her parents. Nevertheless, she expressed ambivalent feelings when it came to different understandings towards certain health beliefs with her husband. Similar to Ayako, Kanako expressed some symptoms of postnatal depression which was mainly due to inter-cultural conflicts with the health professionals and uncertainty in her identity. She has a university degree from Japan and expressed interest in continuing to work in Austria. She expressed great concern about mastering the German language.

Appendix B.

"Consolidated criteria for reporting qualitative research (COREQ): a 32-item checklist for interviews and focus groups" (Tong 2007)

	Items	Question	Answer	Mentioned in
**Domain 1: Research team and reflexivity**				
1	Interviewer	Who conducted the interview?	YS conducted all interviews	2.6. Data collection
2	Credentials	What were the researcher's credentials?	YS: MSc Public Health<br>RS: MSc Social Medicine<br>MK: Professor Public Health	2.7. Data analysis
3	Occupation	What was their occupation at the time of the study?	YS: Lecturer/PhD Student<br>RS: Therapist/PhD Student<br>MK: Professor	2.7. Data analysis
4	Gender	Was the researcher male or female?	Female: YS and RS<br>Male: MK	2.8. Rigor and reflection
5	Experience and training	What experience or training did the researcher have?	YS: Qualitative Methods (QMs)<br>RS: QMs and Counseling<br>MK: Quantitative and QMs in psychology	2.7. Data analysis
Relationships with participants				
6	Relationship established	….prior to study commencement?	Only between YS and Akiko.	2.6. Data collection
7	Participant knowledge of the interviewer	What they knew about the interviewer?	She is an academic researcher doing the research without remuneration	2.5. Ethical consideration
8	Interviewer characteristics	Characteristics reported about the interviewer.	A "Japanese mother, married to an Austrian, given birth in Vienna".	2.6. Data collection
**Domain 2: Study design**				
Theoretical framework				
9	Methodological orientation and Theory	What methodological orientation was stated to underpin the study?	Phenomenology - specifically interpretative phenomenological analysis combined with a deducted approach.	2.1. Design
Participant selection				
10	Sampling	How were participants selected?	Purposive sampling combined with a snowball sampling (getting introduced).	2.4. Sampling and recruitment
11	Method of approach	How were participants approached?	Emails and telephones.	2.4. Sampling and recruitment
12	Sample size	How many participants were there in the study?	Ten (four Chinese-speaking and six Japanese women).	2.4. Sampling and recruitment
13	Non-participation	How many people? Reasons?	Two Chinese-speaking women refused due to time constraints.	2.4. Sampling and recruitment
Setting				
14	Setting of data collection	Where was the data collected?	Participants' home (n=5) and in a park (n=1).	2.6. Data collection
15	Presence of non-participants	Was anyone else present besides the participants and the researcher?	A minor (< 20 months) were present (n=3). Women's spouses were at home but in a different room (n=4).	2.6. Data collection
16	Description of sample	What are the important characteristics of the sample?	Basic demographic data: Table 2.<br>Biographical summary: Appendix A.	3.1. Demographic characteristics<br>Supplementary data
Data collection				

17	Interview guide	Were questions, prompts, guides provided by the author? Was it pilot tested?	Yes, but it was kept to a minimum. The interview guide in Table 1 was not pilot-tested but was used in a systematic and flexible way.	2.6. Data collection
18	Repeat interviews	Were repeat interviews carried out? How many?	Yes, with seven participants (n=7).	2.6. Data collection
19	Audio recording	Was audio or visual recording used?	Yes, 'all interviews were audio recorded with a digital device with consent'.	2.6. Data collection
20	Field notes	Were field notes made during and/or after the interview?	After, to concentrate on the interaction with the participants to do the analysis 'together' in the course of the interview.	2.6. Data collection
21	Duration	What was the duration of the interviews?	From 40 to 115 minutes — average 78 minutes.	2.6. Data collection
22	Data saturation	Was data saturation discussed?	Ten is a sufficient number that meets the objective of our study.	2.4. Sampling and recruitment
23	Transcripts returned	Were transcripts returned to participants for comment and/or corrections?	Yes, the transcripts and later the summary findings were sent back to all participants for comments and corrections.	2.8. Rigor and reflection
**Domain 3: Analysis and findings**				
Data analysis				
24	Number of data coders	How many data coders coded the data?	One. This was rigorously validated by the second author.	2.7. Data analysis
25	Description of the coding tree	Did authors provide a description of the coding tree?	Figure 1.	3.2. Endeavor for gaining trust & respect
26	Derivation of themes	Were themes identified in advance or derived from the data?	Themes were derived from the data but some of the codes used have been identified in advance.	2.7. Data analysis
27	Software	What software, if applicable, was used to manage the data?	Yes, the data analysis software Atlas.ti and Microsoft Excel.	2.8. Rigor and reflection
28	Participant checking	Did participants provide feedback on the finding?	Four participants agreed with the findings. Six only acknowledged that they received it.	2.8. Rigor and reflection
Reporting				
29	Quotations presented	Were participants' quotations presented to illustrate the themes/findings?	Yes. As much as possible significant statements' are provided in the text as well as in the tables.	3. Results<br>Appendix. C
30	Data and findings consistent	Was there consistency between the data presented and the findings?	Yes. We enhanced consistency and transparency by showing each of the analysis process and outcomes in detail.	3. Results
31	Clarity of major themes	Were major themes clearly presented in the findings?	Yes. Especially in Figure 1. in the form of a coding tree.	3. Results
32	Clarity of minor themes	Is there a description of diverse cases or discussion of minor themes?	Yes. We fully described and accounted for the exceptional case.	3. Results<br>4. Discussion

Appendix C. Codebook

Definitions and guidelines used for coding (ensuring inter-coder reliability)

Definitions

Culture: a common systems of knowledge or belief that are learned and internalized through shared practices and repeated interactions among a group of people. A dynamic ideational system that one can refer to as guidance for behavior which is considered appropriate and acceptable in a given cultural context and time.

Acculturative stress: negative emotions experienced by the interviewees who were explicitly or implicitly conscious that such emotions were caused by intercultural interactions and cultural differences. Enculturative stress — a stress caused by interpersonal conflict between people from the same cultural heritage — is also considered as a type of acculturative stress.

Postnatal acculturative stress: acculturative stress experienced immediately after and approximately up to six months after birth in Austria.

Codebook

No.	Code used	Use this code when the meaning unit represents/presents:
**1.**	Cultural practices not understood or respected	the feelings of health beliefs and health practices not understood or respected by the people around.
**2.**	Lack of mutual respect	the interviewee trying to be respectful and thoughtful to the others but feeling not being respected in return.
**3.**	Feeling disrespected for not speaking German	feelings of being disrespected due to not speaking German.
**4.**	Frustrated and irritated	irritation, frustration and anger or the mixture of these emotions.
**5.**	Unexpected and unbelievable	negative surprise, feeling unprepared or encountering something unbelievable, or the mixture of these emotions.
**6.**	Living up to the expectations of unexpected	the struggle of trying to live up to either side of cultural expectations which were not foreseen.
**7.**	Feeling of dependence	the struggle of wanting to be independent but not being able to do so. Closely related to the code: Living up to the expectations of unexpected.
**8.**	Feeling guilty for doing something culturally “inappropriate”	the feeling of shame resulting from the struggle of trying to pursue her cultural practice while being aware that it is not accepted by the host society. Always use with context code (hospital or home).
**9.**	Not wanting to bother people who are not close family members	the feeling of not wanting to be an extra burden to health personnel and being hesitance in seeking proactive support from outside the family members.
**10.**	Postnatal support from people who share the same health beliefs is hard to replace	the feeling that Austrian husband’s and other people support is appreciated but it is not replaceable with that of the support of own family members who the interviewee shares the same cultural heritage and health beliefs.
**11.**	Feeling physical and emotional distance with the family back home	the feeling of loneliness that telephone and sykpe cannot replace the actual physical presence of the family members.
**12.**	Negative emotions due to language/communication barrier	the negative experiences related to language and communication barrier.

**13.**	Diet and constipation	the experiences of differences in health beliefs related to diet and constipation.
**14.**	Diet and breastfeeding	the experiences of differences in health beliefs related to diet and breastfeeding.
**15.**	Seeking external support	the experiences of proactively seeking and receiving support outside the family members.
**16.**	Self-blaming for not being prepared	the expressions of blaming oneself for not being prepared for the (anticipated) differences in how postnatal care is organized in Austria.
	**Career (C) and identity (I)**	
**17.**	C: A career opportunity back home or elsewhere outside Austria	the interviewee's current or past wish in pursing a career opportunity outside Austria.
**18.**	C: giving up career for having a child	the general feeling of the need of giving up (part of) one's career for having a child.
**19.**	C: Not being aware of what having a child in Austria meant to one's career	the feelings of surprise and unexpectedness of not being aware that having a child in Austria was linked with giving up (part of) one's career.
**20.**	I: Not being aware of what having a child meant to one's identity	the feelings of surprise and unexpectedness of not being aware that having a child in Austria was linked with shift in one's identity of becoming a permanent immigrant in Austria.
**21.**	I: Importance of having a social network of own cultural group	the expressions of the recognition of the importance and the needs of having a social network of the interviewee's own cultural group in Austria which became stronger and clearer after birth.
**22.**	I: Accepting the destiny of becoming a permanent immigrant in Austria	the expressions of acceptance and readiness of becoming a permanent immigrant in Austria.
**23.**	I: Providing opportunities for the child to speak the mother-tongue	the interviewee's wish to pass on her cultural identity to her child by providing opportunities for her child to speak Chinese/Japanese.
**24.**	I: Giving the child a Chinese/Japanese name	the interviewee's wish to pass on her cultural identity to her child by given her child a Chinese/Japanese name.
	**Contributing factors to positive/negative experiences**	
**25.**	Lacking confidence	the expressions of lack of confidence in general as well as specific to taking proactive actions.
**26.**	Lacking agency/autonomy	the expressions of not being able to take actions in a way that the interviewee really wanted to.
**27.**	Discovering unexpected negative cultural/acculturative aspects of the significant others	disappointment, negative surprise, and negative discovery expressed by the interviewee in regards to the cultural or acculturative aspects of her husband and parents (especially mother). Always use in combination with the person code.
**28.**	Discovering unexpected positive cultural/acculturative aspects of the significant others	Positive surprise and discovery expressed by the interviewee in regards to the cultural or acculturative aspects of her husband and parents (especially mother). Also use this code when the interviewee expresses she is proud of the openness and the understanding attitudes of her significant others. Always use in combination with the person code.
**29.**	Ambivalent feeling towards own mother/parents	pity, confusion, mixed feeling of appreciation and annoyance expressed by the interviewee in regards to her own mother or parents.
**30.**	Ambivalent feeling towards husband	mixed feeling of appreciation but not being able to fully share the cultural beliefs and practices with her husband.
**31.**	(Dis) Trusting and (dis)respectful relationship with health personnel	stories of interviewee's interactions with Austrian health personnel(s) in which she expresses appreciation, mutual trust and respect or the opposite. Always code together with the personal codes.
**32.**	Trusting and respectful relationship with the	stories of interviewee's interactions with her husband or parents in

	significant others	which she expresses appreciation, mutual trust, respect and sense of pride. Always code together with the personal codes.
**33.**	(Dis)trusting and (Dis)respectful relationship between the significant others	stories and expressions that describe the relationships between her husband and her parents (especially mother) represented by mutual trust, respect and sense of pride, or the lack of these positive elements. Always code together with the personal codes.
**34.**	Trust and pride in own health belief	the interviewee's trust and pride in her home culture's health beliefs and practices. Use the code for the expressions of both high and low trust and pride.
**35.**	Trust in Austrian health belief	the interviewee's trust in Austrian health beliefs and practices. Use the code for the expressions of both high and low trust.
**36.**	Personality: extroversion	interviewee's expressions being extroversive (talkative, optimistic, assertive and cheerful)
**37.**	Personality: introversion	interviewee's expressions being introversive (cynical, pessimistic, depressive or sad)
**38.**	**Context Code:** Apply the context code to clarify the context of the experience	
	38.1. Context: Hospital or public	a setting in the hospital or a public setting
	38.2. Context: Home or private	a setting at home or a private setting
**39.**	**Person Code**: Apply the following person codes when the interviewees talk about them	
	39.1. Person: hospital roommates	
	39.2. Person: nurse	
	39.3. Person: doctor	
	39.4. Person: cleaning lady	
	39.5. Person: private midwife	
	39.6. Person: public midwife	
	39.7. Person: TCM doctor Chinese-speaking	
	39.8. Person: TCM doctor Austrian	
	39.9. Person: Chinese-speaking babysitter	
	39.10. Person: Chinese-speaking friends in Austria	
	39.11. Person: Japanese friends in Austria	
	39.12. Person: Family and friends back home	
	39.13. Person: Family Austrian husband	
	39.14. Person: Family Japanese husband	
	39.15. Person: Family European husband	
	39.16. Person: Family mother	
	39.17. Person: Family father	
	39.18. Person: Family Austrian mother-in-law	
	39.19. Person: Family Austrian father-in-law	
	39.20. Person: Family Japanese mother-in-law	
	39.21. Person: Austrian mothers	
	39.22. Person: Austrian husband's friends	
**40**.	**Domain code:** Apply these domain codes when the meaning unit contains topics related to these five domains.	
	40.1. Postnatal rest	
	40.2. Postnatal diet	

40.3. Social support

40.4. Feeling towards significant others

40.5. Identity

41. Codes developed from Berry's acculturation model

41.1. Confusion	the expressions of losing trust in either side of cultural beliefs, behavior or practices, or all three.
41.2. Proactive assimilation	the interviewee proactively choosing to do the way done in the host society and its people.
41.3. Passive assimilation	the interviewee giving-in to the way done in the host society and its people against her will.
41.4. Conscious separation	the interviewee consciously adhering to her own health belief and practices by secluding herself with the people of her same cultural heritage or by clearly separating the domains in which she follows the 'Austrian-way' or the 'Chinese/Japanese-way' (ex. taking Chinese medicine for chronic symptoms and taking synthetic medicine for acute symptoms).
41.5. Separation as a matter of course	the interviewee adhering to her own health belief and practices as the result of being surrounded by the people of same or similar cultural heritage (intra-nationally married couple, having an East Asian friend as a neighbor, etc.,)
41.6. Integration	the interviewee being able to adhere to her own health practice without secluding and separating herself from the host population as a result of the host population taking part in her practice. It also includes cases of incorporating host country's practice without replacing it with the interviewee's own cultural practice.

Codes summarized into four sub-themes and one major theme and their descriptions

Themes	Descriptions
**Superordinate theme:** Postnatal acculturative stress and coping as unexpected solitary struggles in the midst of identity change	The main theme depicts the experiences of postnatal acculturative stress and coping as something that was unforeseen. Thus the women felt ill-prepared and frustrated. Women did not expect that not being able to share their health beliefs and practices with the host society and people would provoke negative emotions. They also did not expect that not being able to share these complex emotions with their significant others would make them feel isolated and lonely. Women were also surprised by discovering new acculturative aspects of their significant others as well as their own identity of becoming a permanent immigrant mother in a foreign land.
**Subordinate themes A.** **Surprise and irritated:** Surprised and irritated with the consequences of not being able to share health beliefs and practices with the host society and its people	This sub-theme depicts women's irritation, frustration and feeling of unexpectedness for not being able to share health beliefs and practices that are taken for granted at home with the host society and its people.
**Sub-theme B.** **Lonely and isolated:** A lonely and isolated struggle for not being able to share complex emotions and concerns related to cultural health beliefs and practices with the significant others.	This sub-theme depicts women's feeling of loneliness, sadness, and being alone with the struggle of dealing with her complex emotions related to cultural differences. Women felt they could not openly share their feelings with the significant others.
**Sub-theme C.** **Becoming a mother and becoming a permanent immigrant:** Becoming a mother and becoming a 'permanent' immigrant — acculturative stress in a context of a dual identity change	This sub-theme shows that immigrant women go through a change in dual-identity of becoming a permanent immigrant to Austria and becoming a mother to a child born in a country different from hers. On one hand, she is preparing to settle and "assimilate" into the Austrian society and its culture. On the other hand, she becomes aware of the increased needs for supports from her same-cultural group and the willingness to pass on her own cultural heritage to the child (separation).
**Sub-theme D.** **Trust and mutual respect:** Trust and mutual respect with and between the significant others	This sub-theme depicts that positive and negative experiences in the postnatal period are highly influenced by the trust and mutual respectful relationships between the women and their significant others but also between the women's significant others. Respectful interpersonal relationships enabled integrations to happen and prevent and mitigate postnatal stress. Lack of them led to women experiencing unexpected acculturative stress.
